# Candidate Genes Involved in the Biosynthesis of Triterpenoid Saponins in *Platycodon grandiflorum* Identified by Transcriptome Analysis

**DOI:** 10.3389/fpls.2016.00673

**Published:** 2016-05-19

**Authors:** Chun-Hua Ma, Zheng-Jie Gao, Jia-Jin Zhang, Wei Zhang, Jian-Hui Shao, Mei-Rong Hai, Jun-Wen Chen, Sheng-Chao Yang, Guang-Hui Zhang

**Affiliations:** ^1^Yunnan Research Center on Good Agricultural Practice for Dominant Chinese Medicinal Materials, Yunnan Agricultural UniversityKunming, China; ^2^The Life Science and Technology College, Honghe UniversityMengzi, China; ^3^National Engineering Research Center for Agricultural Biodiversity Applied Technology, Yunnan Agricultural UniversityKunming, China

**Keywords:** *Platycodon grandiflorum*, transcriptome, triterpenoid saponins, platycodin D, biosynthesis

## Abstract

**Background:** *Platycodon grandiflorum* is the only species in the genus *Platycodon* of the family Campanulaceae, which has been traditionally used as a medicinal plant for its lung-heat-clearing, antitussive, and expectorant properties in China, Japanese, and Korean. Oleanane-type triterpenoid saponins were the main chemical components of *P. grandiflorum* and platycodin D was the abundant and main bioactive component, but little is known about their biosynthesis in plants. Hence, *P. grandiflorum* is an ideal medicinal plant for studying the biosynthesis of Oleanane-type saponins. In addition, the genomic information of this important herbal plant is unavailable.

**Principal findings:** A total of 58,580,566 clean reads were obtained, which were assembled into 34,053 unigenes, with an average length of 936 bp and N50 of 1,661 bp by analyzing the transcriptome *data* of *P. grandiflorum*. Among these 34,053 unigenes, 22,409 unigenes (65.80%) were annotated based on the information available from public databases, including Nr, NCBI, Swiss-Prot, KOG, and KEGG. Furthermore, 21 candidate *cytochrome* P450 genes and 17 candidate UDP-glycosyltransferase genes most likely involved in triterpenoid saponins biosynthesis pathway were discovered from the transcriptome sequencing of *P. grandiflorum*. In addition, 10,626 SSRs were identified based on the transcriptome data, which would provide abundant candidates of molecular markers for genetic diversity and genetic map for this medicinal plant.

**Conclusion:** The genomic data obtained from *P. grandiflorum*, especially the identification of putative genes involved in triterpenoid saponins biosynthesis pathway, will facilitate our understanding of the biosynthesis of triterpenoid saponins at molecular level.

## Introduction

*Platycodon grandiflorum* (Jacq.) A. DC. is a perennial flowering plant of the Campanulaceae family and the only species of the genus *Platycodon*. It is a well-known medicinal plant in China and other East Asian countries and has been traditionally used as a medicine and food additive for various respiratory diseases, including bronchitis, asthma, tonsillitis, pulmonary tuberculosis and other inflammatory diseases (Takagi and Lee, 1972; Kim et al., 1995; Shin and Lee, 2002). Oleanane-type triterpenoid saponins are the main chemical components of *P. grandiflorum*, mainly including platycodin D, D2, D3, deapioplatycodin D, D2, polygalacin D and platyconic acid A (Kim J.W. et al., 2013). In addition to their natural effects, these triterpenoid saponins have various pharmacological activities, such as anti-inflammatory, anti-cancer, immune enhancing effects and preventing chemicals-induced hepatotoxicity (Lee et al., 2004, 2008; Kim et al., 2008, 2012a; Khanal et al., 2009). Especially, chemical investigation of *P. grandiflorum* has revealed that platycodin D is the most abundant and the main bioactive component (Shin et al., 2009; Xie et al., 2009; Kim et al., 2012b).

**FIGURE 1:**
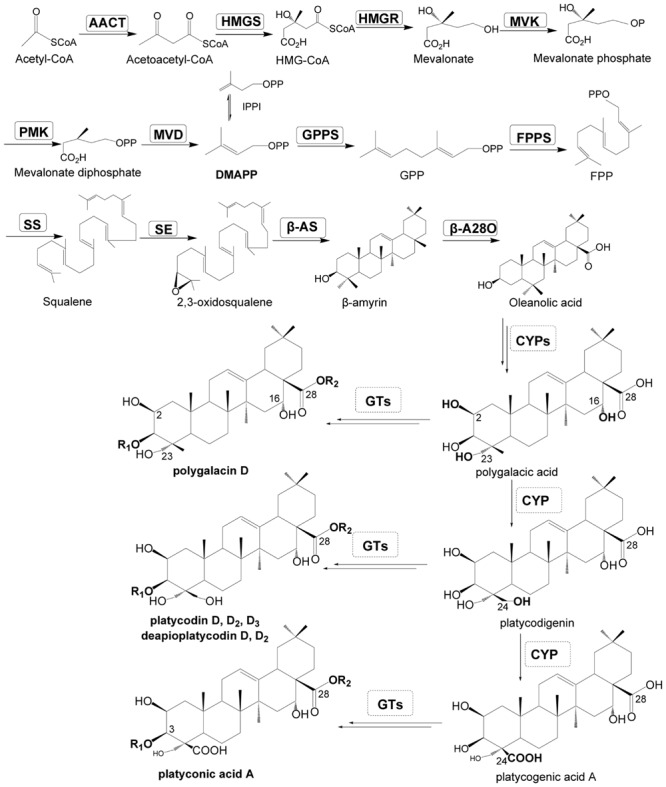
**Putative pathway for triterpenoid saponin biosynthesis in *Platycodon grandiflorum*.** Enzymes found in this study are boxed. AACT, acetyl-CoA acetyltransferase; HMGS, HMG-CoA synthase; IPPI, IPP isomerase; HMGR, HMG-CoA reductase; MVK, mevalonate kinase; PMK, phosphomevalonate kinase; MVD, mevalonate diphosphate decarboxylase; GPPS, geranylgeranyl pyrophosphate synthase; FPPS, farnesyl diphosphate synthase; SS, squalene synthase; SE, squalene epoxidase; β-AS, β-amyrin synthase; β-A28O, β-amyrin 28-oxidase; HMG-CoA, 3-hydroxy-3-methylglutaryl coenzyme A; DMAPP, dimethylallyl diphosphate; FPP, farnesyl diphosphate; GPP, geranyl pyrophosphate; IPP, isopentenyl diphosphate; GT, glycosyltransferase; CYPs, cytochrome P450.

Triterpenoid saponins are a group of mostly studied compounds in plants, and their biosynthesis has been extensively studied and described (Haralampidis et al., 2002; Yendo et al., 2010; Augustin et al., 2011; Moses et al., 2014a). The direct precursor of triterpenoid saponins is 2, 3-oxidosqualene which is synthesized via the mevalonic acid (MVA) pathway (Haralampidis et al., 2002). Three key enzymes are involved in the biosynthesis of these saponins: oxidosqualene cyclases (OSCs), cytochrome P450 monooxygenases (P450s) and uridine diphosphate-dependent glycosyltransferases (UGTs; **Figure 1**, Supplementary Table S4). The most important progress in the biosynthesis of triterpenoid saponins is achieved in *Panax* species (Araliaceae family), which contains a special group of triterpenoid saponins, i.e., ginsenosides. Three P450s in *Panax ginseng* have been functionally characterized, they are protopanaxadiol synthase (PPDS, CYP716A47), which catalyzes the conversion of dammarenediol-II to protopanaxadiol (Han et al., 2011), protopanaxatriol synthase (PPTS, CYP716A53v2) catalyzing the conversion of protopanaxadiol to protopanaxatriol (Han et al., 2012), and β-A28O (CYP716A52v2) catalyzing the conversion of β-amyrin to oleanolic acid (Han et al., 2013). Recently, two UGTs (PgUGT74AE2 and PgUGT94Q2) have also been characterized in *P. ginseng* which are involved in the biosynthesis of ginsenoside Rg3 and Rd (Jung et al., 2014). Even though the biosynthesis of some ginsenosides or their aglycones have been well-documented and can be conducted in a yeast fermentation system (Dai et al., 2014; Jung et al., 2014), the biosynthesis of triterpenoid saponins in different plant species is far from conclusive.

Despite many genes encoding enzymes involved in the biosynthesis of the triterpenoid saponins have been identified from *Panax* species (Sun et al., 2010; Chen et al., 2011; Luo et al., 2011; Li et al., 2013), information about those genes in *P. grandiflorum* is still lacking (Kim Y.K. et al., 2013). Although the pharmacological activity of platycodin D has been investigated (Kim et al., 2012a,b; Chun et al., 2013; Chun and Kim, 2013; Hwang et al., 2013; Li et al., 2014), a complete biosynthesis pathway of platycodin D has not been elucidated, especially the last two steps. At present, the genomes or transcripts of about 46 species of medicinal plants have been sequenced, which will lead to an efficient way of deciphering novel gene functions involved in specific metabolic pathways in medicinal plants (Misra, 2014). Characterization of these novel genes will be useful for investigating the synthesis of platycodins in *P. grandiflorum*. The objective of the present study was to characterize the transcriptome of *P. grandiflorum* using Illumina HiSeq^TM^2000 sequencing platform in order to uncover the candidate genes encoding enzymes involved in the triterpene saponin biosynthetic pathway, especially in oleanane-type saponins biosynthesis, and to screen molecular markers of SSRs for facilitation the marker-assisted breeding of this species.

## Results and Discussion

### Illumina Sequencing and *De Novo* Assembly

The root tissue of *P. grandiflorum* was used for transcriptome sequencing and analysis because roots have traditionally been used for medicinal purpose. A cDNA library was constructed from total RNA of *P. grandiflorum* roots, and sequenced using Illumina paired-end sequencing technology. After removal of adaptor sequences, ambiguous reads and low-quality reads (Q20 < 20), a total of 58,580,566 clean reads were obtained. The Q20 percentage (sequencing error rate < 1%) and GC percentage were 97.04 and 45.51%, respectively. An overview of the sequencing and assembly statistics is shown in **Table 1**. The high quality reads obtained in this study have been deposited in the NCBI SRA database (accession number: SRA226668).

**Table 1:** Summary of Illumina paired-end sequencing and assembly for *Platycodon grandiflorum.*

Database	Number	Total length (bp)
Total clean reads	58,580,566	58,580,56600
Q20 percentage	97.04%	
GC percentage	45.51%	
Number of transcripts	50,408	55,568,306
Average length of transcripts (bp)	1,102	
Max length of transcripts (bp)	15,684	
Min length of transcripts (bp)	201	
Transcript size N50 (bp)	1,796	
Number of unigenes	34,053	31,887,854
Average length of unigenes (bp)	936	
Max length of unigenes (bp)	15,684	
Min length of unigenes (bp)	2,01	
Unigene size N50 (bp)	1,661	

**FIGURE 2:**
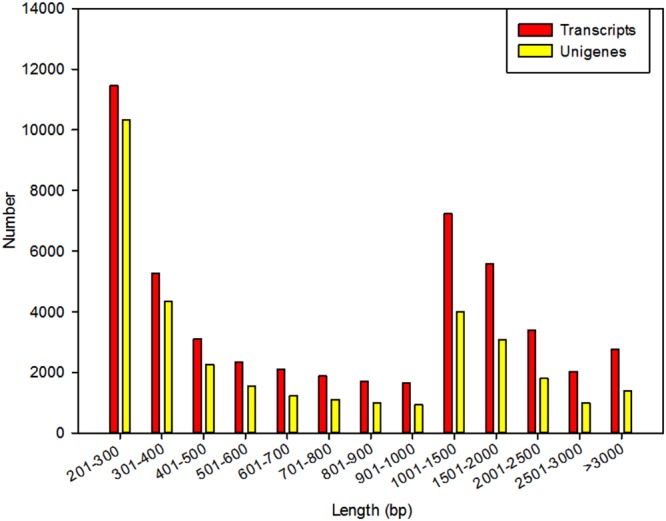
**Overview of the *P. grandiflorum* transcriptome assembly**.

All the clean reads (58,580,566) were *de nov*o assembled using the Trinity program into 50,408 transcripts consisting of 55,568,306 bp. The size of the transcripts ranged from 201 to 15,684 bp, with an average length of 1,102 bp and N50 length of 1,796 bp. Among these transcripts, 20,939 (41.54%) were longer than 1000 bp, and 19,808 (39.30%) were shorter than 500 bp (**Figure 2**). Using paired-end joining and gap-filling methods, these contigs were further assembled into 34,053 unigenes with an average length of 936 bp and an N50 length of 1,661 bp. There were 11,291 unigenes (33.16%) longer than 1,000 bp, and 4,202 unigenes (12.34%) longer than 2,000 bp (**Figure 2**).

### Functional Annotation

In our study, we used the Nr, Nt, KEGG, SwissProt, PFAM, GO, and KOG publicly available databases to annotate the unigenes. The overall function annotation is depicted in **Table 2**. Altogether, 22,409 unigenes (65.80%) were annotated in the public databases. There were 21,310 unigenes (62.57%) matched in the Nr databases, and 11,877 unigenes (34.87%) matched with known proteins in the Nt databases. A total of 6,998 unigenes (20.55%) matched to the KEGG database and 15,870 unigenes (46.60%) matched to the SwissProt. The number of unigenes matched to the PFAM, GO and KOG databases was 14,877 (43.68%), 16,677 (48.97%), and 8,779 (25.78%), respectively.

**Table 2:** Summary of the annotation percentage of *P. grandiflorum* compared with public databases.

Database	Number of unigenes	Annotation percentage (%)
Nr	21,310	62.57
Nt	11,877	34.87
KEGG	6,998	20.55
SwissProt	15,870	46.60
PFAM	14,877	43.68
GO	16,677	48.97
KOG	8,779	25.78
All annotated unigenes	22,409	65.80
Total unigenes	34,053	

### Gene Ontology Classification

**FIGURE 3:**
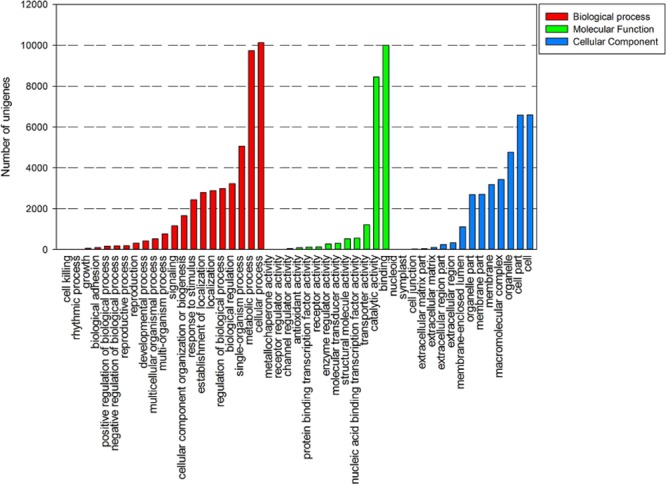
**Gene ontology classification of assembled unigenes**.

A total of 16,677 unigenes were characterized using GO analysis based on Nr annotation, including biological process, cellular component, and molecular function. There were 31,810 unigenes were grouped under cellular component, 21,705 unigenes under molecular function, 44,810 unigenes under biological process. Under the cellular component category, the majority of unigenes were involved in cell (6,586 unigenes, 20.29%) and cell part (6,579 unigenes, 20.27%). For the biological process class, the cellular process (10,127 unigenes, 22.50%) and metabolic process (9,737 unigenes, 21.63%) were the most abundant classes. In the molecular function category, binding (9,999, 46.07%) and catalytic activities (8,438, 38.88%) were predominant (**Figure 3**).

### KOG Classification

**FIGURE 4:**
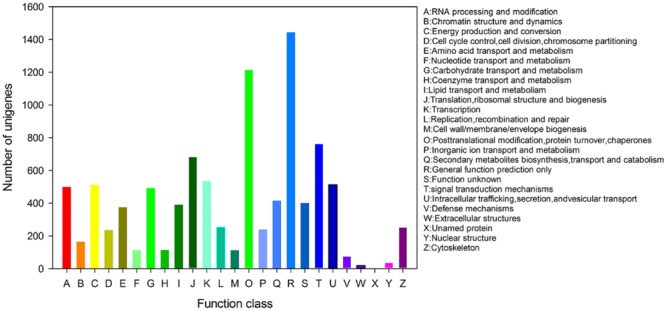
**Eukaryotic Orthologous Groups (KOGs) function classification of *P. grandiflorum***.

All unigenes were subjected to a search against the KOG database for functional prediction and classification. Totally, 8,779 unigenes were clustered into 26 functional categories. The general function prediction only (1,444 unigenes, 16.45%) was the major KOG category, followed by post-translational modification, protein turnover, chaperones (1,215 unigenes, 13.84%), signal transduction mechanisms (763 unigenes, 8.7%), translation, ribosomal structure and biogenesis (683 unigenes, 7.78%), transcription (534 unigenes, 6.08%), intracellular trafficking, secretion, and vesicular transport (513 unigenes, 5.84%), energy production and conversion (512 unigenes, 5.83%; **Figure 4**).

### Functional Classification by KEGG

**FIGURE 5:**
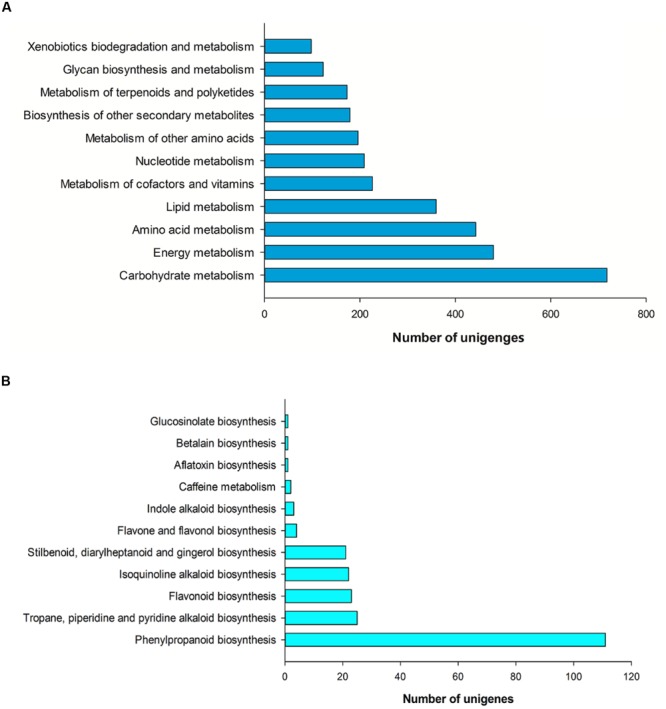
**Pathway assignment based on KEGG. (A)** Classification based on metabolism categories. **(B)** Classification based on biosynthesis of other secondary metabolites.

In our study, 6,998 unigenes (20.55%) were annotated and assigned to 258 pathways by the KEGG, including metabolism, genetic information processing, environmental information processing, cellular processes, organismal systems and human diseases. The category with the largest number of unigenes was metabolism, which included carbohydrate metabolism (718 unigenes, 19.43%), energy metabolism (480 unigenes, 12.99%), amino acid metabolism (443 unigenes, 11.99%), lipid metabolism (360 unigenes, 9.74%), metabolism of cofactors and vitamins (226 unigenes, 6.11%), nucleotide metabolism (209 unigenes, 5.65%), metabolism of other amino acids (196 unigenes, 5.30%), biosynthesis of other secondary metabolites (179 unigenes, 4.84%), metabolism of terpenoids and polyketides (173 unigenes, 4.68%), glycan biosynthesis and metabolism (123 unigenes, 3.33%), xenobiotics biodegradation and metabolism (98 unigenes, 2.65%; **Figure 5A**).

In the other secondary metabolites, the most represented category was phenylpropanoid biosynthesis (111 unigenes, 62.01%), followed by tropane, piperidine and pyridine alkaloid biosynthesis (25 unigenes, 13.97%), flavonoid biosynthesis (23 unigenes, 12.85%), isoquinoline alkaloid biosynthesis (22 unigenes, 12.29%), stilbenoid, diarylheptanoid and gingerol biosynthesis (21 unigenes, 11.73%), flavone and flavonol biosynthesis (4 unigenes, 2.23%; **Figure 5B**).

### Candidate Genes Encoding Enzymes Involved in Triterpenoid Saponin Biosynthesis

The transcripts encoding all the known enzymes involved in triterpenoid saponin biosynthesis were discovered in this Illumina dataset, including AACT, HMGS, HMGR, MVK, PMK, MVD, GGPPS, FPPS, IPPI, SS, SE, β-AS, and β-A28O (**Table 3**). These findings were in accordance with the fact that *P. grandiflorum* contains high contents of oleanane-type saponins. Platycodin D is the main triterpenoid saponin in *P. grandiflorum*, the β-AS (seven unigenes) and β-A28O (one unigenes) were the key enzymes in the biosynthesis of platycodin D. Functional characterization of these unigenes will help us to understand the molecular mechanism of the biosynthesis of oleanane-type saponins in *P. grandiflorum*.

**Table 3:** Transcripts involved in triterpene saponin biosynthesis pathway in *P. grandiflorum.*

Gene name	EC number	Unigene number
AACT, acetyl-CoA acetyltransferase	2.3.1.9	2
HMGS, hydroxymethylglutaryl-CoA synthase	2.3.3.10	5
HMGR, hydroxymethylglutaryl-CoA reductase	1.1.1.34	4
MVK, mevalonate kinase	2.7.1.36	3
PMK, phosphomevalonate kinase	2.7.4.2	2
MVD, mevalonate diphosphate decarboxylase	4.1.1.33	1
GPPS, geranylgeranyl pyrophosphate synthase	2.5.1.29	6
FPPS, farnesyl diphosphate synthase	2.5.1.10	1
IPPI, isopentenyl diphosphate isomerase	5.3.3.2	1
SS, squalene synthase	2.5.1.21	1
SE, squalene epoxidase	1.14.99.7	10
β-AS, β-amyrin synthase	5.4.99.39	7
β-A28O, β-amyrin 28-oxidase	1.14.13.-	1

### The Cytochrome P450 Monooxygenases and UDP-Glycosyltransferase Genes

**FIGURE 6:**
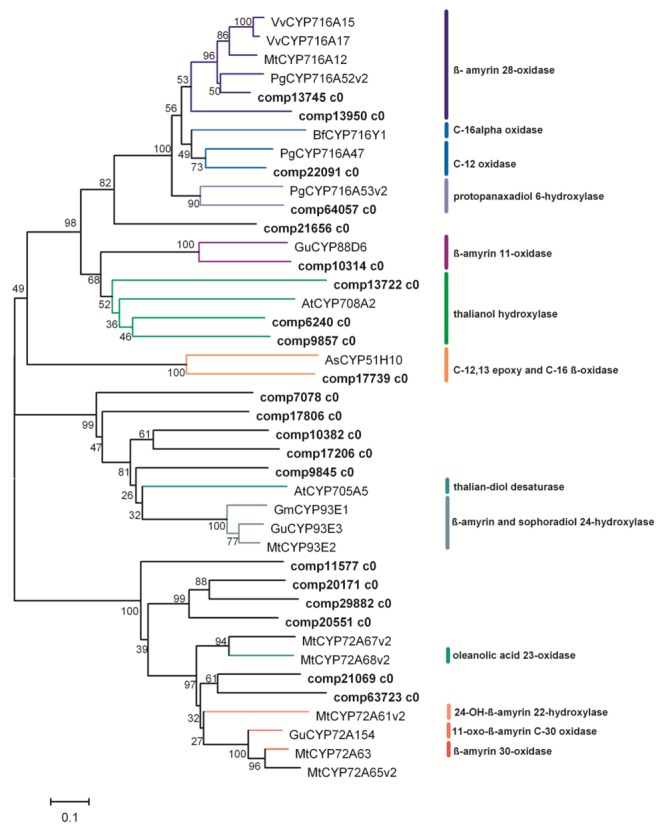
**Phylogenetic tree of the *P. grandiflorum* CYP450s.** Phylogenetic tree is constructed based on the deduced amino acid sequences for the *P. grandiflorum* CYP450s (bold letters) and other plant CYP450s involved in triterpenoid biosynthesis. Protein sequences are retrieved from NCBI GenBank using the following accession numbers: *Vitis vinifera* VvCYP716A15 (BAJ84106.1) and VvCYP716A17 (BAJ84107.1); *Medicago truncatula* MtCYP716A12 (ABC59076.1), MtCYP93E2 (ABC59085), MtCYP72A63 (H1A981.1), MtCYP72A65v2 (BAL45202), MtCYP72A67v2 (BAL45203) and MtCYP72A67v2 (BAL45203), and MtCYP72A61v2 (BAL45199); *Panax ginseng* PgCYP716A52v2 (AFO63032.1), PgCYP716A53v2 (I7CT85.1) and PgCYP716A47 (H2DH16.2); *Arabidopsis thaliana* AtCYP708A2 (NP_001078732.1) and AtCYP705A5 (EFH40098); *Glycyrrhiza uralensis* GuCYP88D6 (B5BSX1.1), GuCYP93E3 (BAG68930) and GuCYP72A154 (H1A988.1); *Avena strigosa* AsCYP51H10 (ABG88965.1); *Glycine max* GmCYP93E1 (NP_001236154.1); BfCYP716Y1.

The CYP450 enzymes, which catalyze the oxidations of β-amyrin, especially at C-2, C-16, C-23, C-24 and C-28, are required for the biosynthesis of the main triterpenoid saponins in *P. grandiflorum* (**Figure 1**). In the transcriptomic data of *P. grandiflorum*, 87 unigenes were annotated to CYP450 (Supplementary Table S1). Among them, unigene comp13745 c0 was annotated to *P. ginseng* CYP716A52v2 (**Figure 6**), and com13950 c0 was highly homologous to *P. ginseng* CYP716A52v2, *Medicago truncatula* CYP716A12, *Vitis vinifera* CYP716A15 and CYP716A17 (Carelli et al., 2011; Fukushima et al., 2011; Han et al., 2013), strongly suggesting that both of them might encode β-A28O catalyzing conversion of β-amyrin to oleanolic acid (**Figure 6**). *Bupleurum falcatum* CYP716Y1 catalyzes conversion of β-amyrin to 16α hydroxyl β-amyrin (Moses et al., 2014b), no homologous gene was found in this study, and only one unigene (comp21656 c0) was of some similarity (**Figure 6**). Two unigenes (comp21069 c0 and comp63723 c0) were homologous to *M. truncatula* CYP72A68v2 which catalyze the hydroxylation of oleanolic acid at C-23 (Fukushima et al., 2013), suggesting that both of them have the same catalytic activities in *P. grandiflorum*. We also found that five unigenes (comp7080 c0, comp17806 c0, comp10382 c0, comp17206 c0, and comp9845 c0) were highly homologous to CYP93E1 of *Glycine max, M. truncatula*, and *Glycyrrhiza uralensis* (Seki et al., 2008), which catalyzes the C-24 hydroxylation of β-amyrin and sophoradiol in soyasaponin biosynthesis (Shibuya et al., 2006; Li et al., 2007; Seki et al., 2008; Fukushima et al., 2013), thus proteins encoded by these unigenes might be also responsible for the C-24 hydroxylation in *P. grandiflorum*.

Surprisingly, two unigenes (comp22091 c0 and comp64057 c0), which were highly homologous to *P. ginseng* CYP716A47 (Han et al., 2011) and CYP716A53v2 (Han et al., 2012), were also found in these transcriptomic data, suggesting that trace amount of protopanaxadiol-type and protopanaxatriol-type ginsenosides might also be synthesized in the root of *P. grandiflorum*. Moreover, some unigenes homologous to *G. uralensis* CYP88D6 (β-amyrin 11-oxidase, Seki et al., 2008), *Avena strigosa* CYP51H10 (C-12, 13 epoxy and C-16 β-oxidase, Qi et al., 2006; Kunii et al., 2012; Geisler et al., 2013) and *Arabidopsis* CYP708A2 (thalianol hydroxylase) and CYP705A5 (thaliana-diol desaturase) were also found in the transcriptomic data of *P. grandiflorum* (Field et al., 2011). In the putative pathway, we proposed that the carboxylation at C-28 is before the hydroxylation reactions at other carbon atoms (**Figure 1**); actually it is more likely to occur in the opposite order. Even though some unigenes are homologous to the known CYP450s in other plants, further studies are needed to characterize their functions in the biosynthesis pathway of triterpenoid saponins, including those key intermediates in *P. grandiflorum.*

**FIGURE 7:**
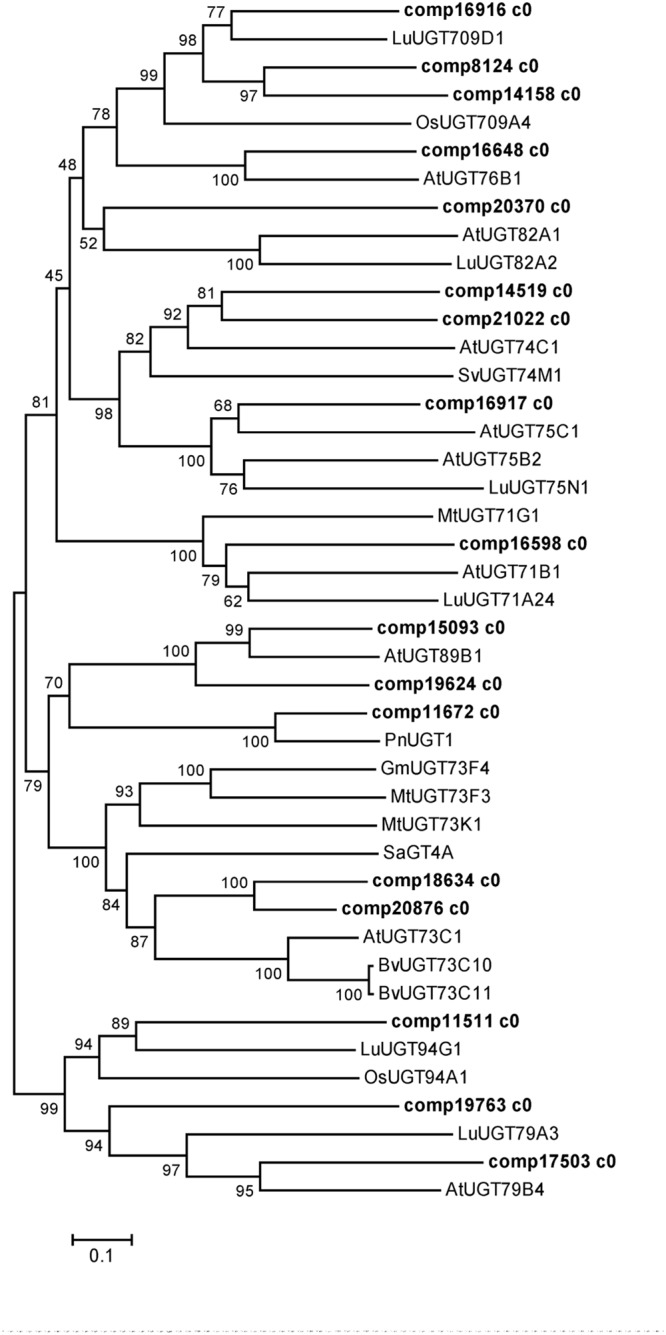
**Phylogenetic tree constructed based on the deduced amino acid sequences for the *P. grandiflorum* UGTs (bold letters) and other plant UGTs.** Accession numbers in the NCBI GenBank database are as follows: *Barbarea vulgaris* BvUGT73C11 (AFN26667) and BvUGT73C10 (AFN26666); *Arabidopsis thaliana* AtUGT73C1 (NP_181213.1), AtUGT82A1 (NP_188864.1), AtUGT76B1 (NP_187742.1), AtUGT71B1 (NP_188812.1), AtUGT89B1 (NP_177529.2), AtUGT75B2 (NP_172044.1), AtUGT75C1 (NP_193146.1), AtUGT74C1 (NP_180738.1), AtUGT79B4 (Q9LJA6.1) and AtUGT79B1 (Q9LVW3.1); *Solanum aculeatissimum* SaGT4A (BAD89042); *M. truncatula* MtUGT73K1 (AAW56091), MtUGT73F3 (ACT34898) and MtUGT71G1 (AAW56092); *G max* GmUGT73F4 (BAM29363); *Panax notoginseng* PnUGT1 (JX018210); *Oryza sativa* OsUGT709A4 (Q7XHR3); *Saponaria vaccaria* SvUGT74M1 (ABK76266); *Linum usitatissimum* LuUGT71A24 (AFJ52909), LuUGT82A2 (AFJ52979), LuUGT709D1 (AFJ53007), LuUGT75N1 (AFJ52962), LuUGT94G1 (AFJ53037.1), LuUGT79A3 (AFJ52973.1).

Uridine diphosphate-dependent glycosyltransferases catalyze the glucosylation of C3- and C28-carboxyl for the biosynthesis of triterpenoid saponins in *P. grandiflorum* (**Figure 1**). In the present study, 106 unigenes encoding UGTs were obtained (Supplementary Table S2), the phylogenetic relationship between these UGTs and characterized UGTs from other plants is depicted in **Figure 7**. Two unigenes (comp18634 c0 and comp20876 c0) were highly homologous to *Barbarea vulgaris* UGT73C11 and UGT73C10, which catalyze sapogenin 3-*O*-glucosylation (Augustin et al., 2012), suggesting that both of them have the same function in *P. grandiflorum*. Two unigenes (comp18634 c0 and comp20876 c0) were closely related to *Saponaria vaccaria* UGT74M1, which is a triterpene carboxylic acid glucosyltransferase (Meesapyodsuk et al., 2007), suggesting that these two unigenes might catalyze the glucosylation of C28-carboxyl for the biosynthesis of triterpenoid saponins. Further studies are required to characterize functionally the aforementioned four unigenes in the biosynthesis of triterpenoid saponins in *P. grandiflorum*.

### Tissue-Specific Expression of Genes Involved in the Biosynthesis of Triterpenoid Saponins

**FIGURE 8:**
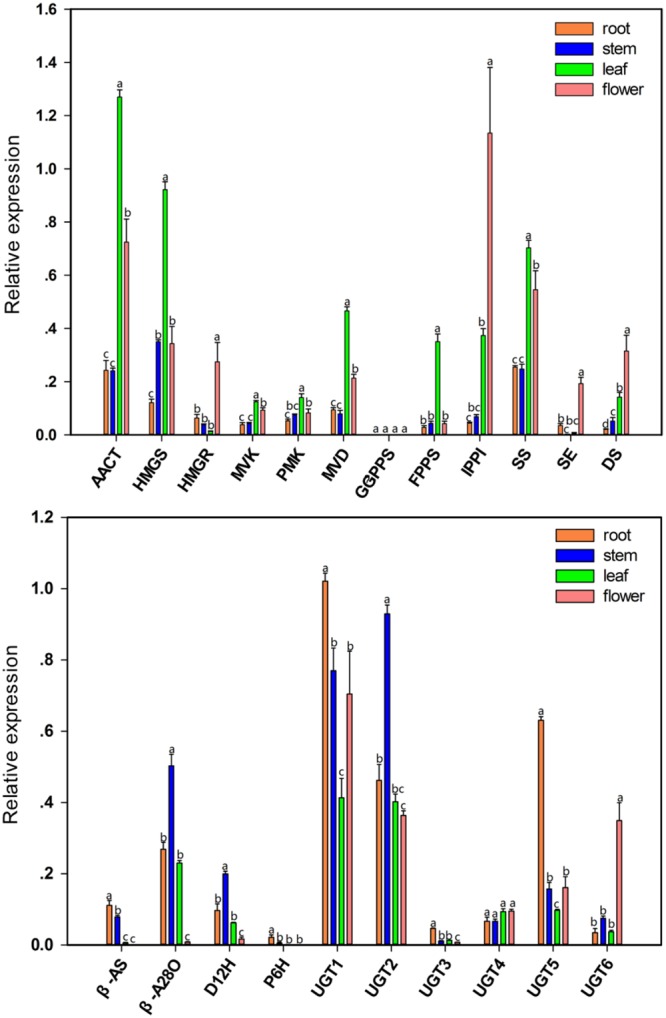
**Validation of candidate unigenes involved in triterpene saponin biosynthesis in *P. grandiflorum* by qPCR.** Bars represent the mean (± SD) of four experiments. Statistical analysis is performed with one way ANOVA with Tukey’s test to compare the difference in the mean expression level of a given gene among different tissues. *P* ≤ 0.05 was considered statistically significant.

The qPCR analysis was used to investigate the tissue-specific expression patterns of 19 unigenes related to the triterpenoid saponin biosynthesis in this species. The expression pattern of these genes is shown in **Figure 8**. The unigenes encoding AACT, HMGS, MVK, PMK, MVD, FPPS, and SS were expressed at much higher level in leaves than in roots, young stems, and flowers (*P* < 0.05). The HMGR, IPPI, and SE genes showed very high expression in the flower tissue (*P* < 0.05). All genes mentioned above play a role in upstream biochemical reactions of the triterpenoid saponin pathway, and showed high expression at mRNA level in leaves and flowers, indicating that leaves are the factories for synthesizing the precursors of triterpenoid saponins. A high expression of β-A28O was observed in young stems (*P* < 0.05), but PD accumulated mainly in roots, indicating that young stems were the modification site of triterpenoid saponins before storage. UGT1 and UGT5 were expressed at much higher level in roots than in other tissues (*P* < 0.05), whereas the expression level of UGT1 and UGT2 was higher in *P. grandiflorum* as compared to that of UGT3, UGT4, UGT5, and UGT6. These results demonstrated that the expression of several genes involved in the biosynthesis of triterpenoid saponins in *P. grandiflorum* was in a tissue-specific manner.

### SSR Marker Analysis

In order to develop SSR markers in *P. grandiflorum*, MISA software was used to detect the SSRs in 34,053 unigenes. A total of 10,626 SSRs were identified in 8,185 unigenes. Among them, 1,916 sequences contained more than one SSR and 807 SSRs were found in compound formation. On average, 3.33 SSRs per 10 Kb were found. In 10,626 SSRs identified the di-nucleotide repeat motifs were the most abundant types (46.05%), followed by mono (33.99%), tri- nucleotide (17.79%), tetra-nucleotide (1.77%), penta-nucleotide (0.24%), and hexa-nucleotide tandem repeats (0.16%; **Tables 4** and **5**).

**Table 4:** Summary of SSR markers.

Item	Number
Total number of sequences examined	34,053
Total size of examined sequences (bp)	31,887,854
Total number of identified SSRs	10,626
Number of SSR containing Sequences	8,185
Average number of SSRs per 10 kb	3.33
Number of sequences containing more than 1 SSR	1,916
Number of SSRs present in compound formation	807

**Table 5:** Distribution of identified SSRs using the MISA software.

Motif	Repeat numbers							Total	%
	5–8	9–12	13–16	17–20	21–24	25–28	29–33		
Mono-	0	1860	835	697	220	0	0	3612	33.99
Di-	2866	2027	0	0	0	0	0	4893	46.05
Tri-	1885	2	2	0	0	0	1	1890	17.79
Tetra-	185	3	0	0	0	0	0	188	1.77
Penta-	25	1	0	0	0	0	0	26	0.24
Hexa-	14	2	0	1	0	0	0	17	0.16
Total	4975	3895	837	698	220	0	1	10626	100.00
%	46.82	36.66	7.88	6.57	2.07	0.00	0.01	100.00	

## Conclusion

Transcriptome sequencing of *P. grandiflorum* was performed for the first time using Illumina next-generation sequencing technologies and a total of 34,053 unigenes were obtained. Particularly, 19 unigenes involved in the biosynthesis of triterpenoid saponins were identified, the expression of which was in a tissue-specific manner. These findings will not only provide valuable information for our complete understanding of the biosynthesis pathway of triterpenoid saponins in *P. grandiflorum*, but also provide opportunities for the *de novo* production of active ingredients by engineering microorganisms. Furthermore, this study will also contribute to the improvements on this species through marker-assisted breeding or genetic engineering.

## Materials and Methods

### Ethics Statement

No specific permits were required for the described field studies. No specific permissions were required for these locations and activities. The location was not privately owned or protected in any way and the field studies did not involve endangered or protected species.

### Plant Materials

Two-years-old *P. grandiflorum* plants were collected from Jianchuan County, Yunnan province, southwest of China (Latitude: 26° 16′ 13″ N, Longitude: 99° 32′ 4″ E, Altitude: 2900 m). After morphological and molecular identification according to the reference (Kim et al., 2012a), the root tissues were collected, frozen immediately in liquid nitrogen, and stored at -80°C until use.

### RNA Library Preparation and Sequencing

Total RNA was extracted from roots by using Trizol reagent (Invitrogen), following by purification with RNeasy MiniElute Cleanup Kit (Qiagen) according to the manufacture’s protocol. For mRNA library construction and deep sequencing, at least 20 μg of total RNA samples were prepared by using the NEBNext^®^ Ultra^TM^ RNA Library Prep Kit for Illumina sequencing on Hiseq 2000 platform at Novogene Bioinformatics Technology, Co. Ltd., (Beijing, China). The high quality reads obtained in this study have been deposited in the NCBI SRA database.

### Transcriptome Data Processing and Assembly

The raw data processing was the same as described previously (Zhang et al., 2015). In brief, raw reads with adaptors and unknown nucleotides above 5% or those that were of low quality (containing more than 50% bases with Q-value ≤ 20) were firstly removed to obtain clean reads using a custom Perl script. Then the clean reads were *de novo* assembled using Trinity program (K-mer = 25, group pairsdistance = 300) with default parameters (Grabherr et al., 2011). Firstly, clean reads with a certain length of overlap were combined to form longer fragments without N, which were called contigs. These clean reads were then mapped back to the corresponding contigs with paired-end reads to detect contigs from the same transcript as well as the distances between contigs, and their paired-end information was also used to fill gaps or extend the sequences. Finally, these resultant sequences were clustered to remove redundant sequences using the TIGR gene Indices clustering tools (TGICL) to form longer sequences without N and cannot be extended on either end. Such sequences are defined as unigenes.

### Functional Annotation and Predicted CDS

Functional annotations were performed as described previously (Zhang et al., 2015). Briefly, functional annotations were performed by sequence comparison with public databases, including the NCBI non-redundant nucleotide database^1^, non-redundant protein database, Swiss-Prot database^2^ and the KOG database using BLASTN and BLASTX^3^, with an e-value of 1e-5. A Perl script was written to assign the functional class to unigenes. Unigenes were also compared with KEGG (Kanehisa et al., 2006) using BLASTX with an e-value of less than 1e-10. A Perl script was used to retrieve KEGG Orthology (KO) information from blast result and then established pathway associations between unigenes and database. Based on the results of Nr database annotation, we used Blast2GO program (Conesa et al., 2005) to perform GO annotation of unigenes. After achieving GO annotation for every unigene, WEGO (Ye et al., 2006) software was used to perform GO classification and draw GO tree. Moreover, the conserved domains/families of the assembled unigenes encoding proteins were searched against the Pfam database (version 26.0; Finn et al., 2014) using Pfam_Scan script.

The CDS for unigene was predicted by BlastX and ESTscan. The unigene sequences were searched against the Nr, KOG, KEGG, and Swiss-Prot protein databases using BLASTX (e-value < 10-5). Unigenes aligned to a higher priority database would not be aligned to lower priority database. The best alignment results were used to determine the sequence direction of unigenes. When a unigene could not be aligned to any database, ESTScan (Iseli et al., 1999) program was used to predict coding regions and determine sequence direction.

### EST-SSR Detection and Primer Design

Potential SSR markers were detected among the 34,053 unigenes using the MISA tool^4^ as described previously (Jiang et al., 2014). We searched for SSRs with motifs ranging from mono- to hexa-nucleotides in size. The minimum of repeat units were set as follows: 10 repeat units for mono-nucleotide, six for di-nucleotides, and five for tri-, tetra-, penta-, and hexa-nucleotides. Primer pairs were designed using Primer3^5^ with default parameters.

### Phylogenetic Analysis

Phylogenetic analysis was performed based on the deduced amino acid sequences of CYP450 and UGT from *P. grandiflorum* and other plants. All of the deduced amino acid sequences were aligned with Clustal X with a gap opening penalty of 10, a gap extension penalty of 0.1, a delay divergent cutoff of 25%, and the other default parameters as described previously (Jiang et al., 2014). The evolutionary distances were computed using MEGA5.10 with the Poisson correction method. For the phylogenetic analysis, a neighbor-joining tree was constructed using MEGA5.0. Bootstrap values obtained after 1000 replications are indicated on the branches. The scale represents 0.1 amino acid substitutions per site.

### Quantitative Real-Time PCR (qPCR) Analysis

Nineteen unigenes with potential roles in ginsenoside biosynthesis were chosen for validation using qPCR with gene specific primers designed with Primer3 software, as described previously (Zhang et al., 2015). All the primer sequences used for the qPCR analysis are shown in Supplementary Table S3. Total RNA from different organs (roots, stems, leaves, and flowers) of *P. grandiflorum* were extracted individually using Trizol Kit (Promega, USA) following the manufacturer’s protocol. Subsequently, RNA was treated with 4 × g DNA wiperMix at 42°C for 2 min to remove DNA. The purified RNA (1 μg) was reverse transcribed to cDNA using HiScript QRT SuperMix for qPCR (Vazyme, Nanjing, China). The qPCR reactions were performed in a 20 μl volume composed of 2 μl of cDNA, 0.4 μl of each primer, and 10 μl 2 × SYBR Green Master mix (TaKaRa) in Roche LightCycler 2.0 system (Roche Applied Science, Branford, CT, USA). 574 PCR amplifications were performed under the following conditions: 30 s at 94°C, followed by 45 cycles of 94°C for 20 s, 55°C for 20 s, and 72°C for 30 s. Three technical replications were performed for all qPCRs. The PMK gene, which was found in our transcriptome database, was chosen as reference control for normalization after the expression of three reference genes (actin, GAPDH, and PMK) was compared in different tissues. The relative changes in gene expression levels were calculated using the 2^-ΔΔCt^ method. For a given gene, the relative expression level was expressed as mean ± standard deviation (SD) of three determinations after normalization with the mRNA level of reference gene PMK. One way ANOVA with Tukey’s test was used to compare the difference in the mean expression level of a given gene among different organs. *P* ≤ 0.05 was considered statistically significant.

## Author Contributions

This study was conceived by G-HZ and S-CY. The plant material preparation were carried out by M-RH and J-HS. Z-JG, J-JZ, and WZ analyzed the RNA-Seq data. C-HM and G-HZ drafted the manuscript. J-WC and C-HM revised the manuscript. All authors read and approved the final manuscript.

## Conflict of Interest Statement

The authors declare that the research was conducted in the absence of any commercial or financial relationships that could be construed as a potential conflict of interest.

## Footnotes

1 http://www.ncbi.nlm.nih.gov/

2 http://www.expasy.ch/sprot

3 http://blast.ncbi.nlm.nih.gov/Blast.cgi

4 http://pgrc.ipk-gatersleben.de/misa/

5 http://bioinfo.ut.ee/primer3-0.4.0/primer3/

## Abbreviations

β-A28O: β-amyrin 28-oxidase
β-AS: β-amyrin synthase
AACT: acetyl-CoA acetyltransferase
Api: apiose
Ara: arabinose
BLAST: Basic Local Alignment Search Tool
bp: base pair
cDNA: complementary DNA
CDS: coding sequence
CYPs: cytochrome P450
DMAPP: dimethylallyl diphosphate
FPP: farnesyl diphosphate
FPPS: farnesyl diphosphate synthase
Gen: gentiobiose
Glc: glucose
GO: Gene Ontology
GPP: geranyl pyrophosphate
GPPS: geranylgeranyl pyrophosphate synthase
GT: glycosyltransferase
HMG-CoA: 3-hydroxy-3-methylglutaryl coenzyme A
HMGR: HMG-CoA reductase
HMGS: HMG-CoA synthase
IPP: isopentenyl diphosphate
IPPI: IPP isomerase
KEGG: Kyoto Encyclopedia of Genes and Genomes
KOGs: Eukaryotic Orthologous Groups
Lam: lipoarabinomannan
MVD: mevalonate diphosphate decarboxylase
MVK: mevalonate kinase
NCBI: National Center for Biotechnology Information
Nr: non-redundant protein
PMK: phosphomevalonate kinase
Rha: Rhamnose
SE: squalene epoxidase
SS: squalene synthase
SSRs: simple sequence repeats
Xyl: xylose
