# Supplementary material for: Candidate Genes Involved in the Biosynthesis of Triterpenoid Saponins in Platycodon grandiflorum Identified by Transcriptome Analysis
Source: Front Plant Sci. 2016 May 19;7:673. doi: 10.3389/fpls.2016.00673 (PMC4871891; doi:10.3389/fpls.2016.00673)
Supplement: Supplementary file 3 [file Table_3.DOC]

Additional file 3. Primers used for gene expression analysis by quantitative real-time PCR (qPCR)

| **Primer name** | **Sequence** |
| --- | --- |
| AACT-F | 5’-CCTCAATACCCCCAAGAGTGTC-3’ |
| AACT-R | 5’-AATGAAGCCTTTGCTGTCGTC-3’ |
| HMGS-F | 5’-GAATCTGGAAATACTGACATTGAAGG-3’ |
| HMGS-R | 5’-CCCTCAGCATAGACCGCACT-3’ |
| HMGR-F | 5’-GGTGGGCGATAATCAAACTAACTC-3’ |
| HMGR-R | 5’-CGAGGATGAATCGTGTGACTGT-3’ |
| MVK-F | 5’-CCTTTAGCATCATCATTTCGCA-3’ |
| MVK-R | 5’-CCATACTCTAAAACTGTTGTTCGCTA-3’ |
| PMK-F | 5’-ATCGTTGGCAGCCCTTCC-3’ |
| PMK-R | 5’-CCAGTCTTTGCTACTTCAGGCTT-3’ |
| MVD-F | 5’-ACATCTCCTTTGGATTTCTGCG-3’ |
| MVD-R | 5’-CTTCAGAGGCTGCTTTTTCACTT-3’ |
| GPPS-F | 5’-CCCACCAGCCATAAAGTCTACG-3’ |
| GPPS-R | 5’-CTGCTACTAAGCCTTCCGTTCC-3’ |
| FPPS-F | 5’-GCTACAAGTTGCTGAAAGGAGAAGA-3’ |
| FPPS-R | 5’-GGTTGACCTCGGCGTGTATG-3’ |
| IPPI-F | 5’-GCAACTTCGTCGGGATTCG-3’ |
| IPPI-R | 5’-TGCTCTACAAGGCACCATCTGAC-3’ |
| SS-F | 5’-CGGATGATTTCTACCCGTTGTT-3’ |
| SS-R | 5’-CTGTTGAATAACGAGGGCGAAG-3’ |
| SE-F | 5’-CACCACGACTTCTATCAACGGA-3’ |
| SE-R | 5’-GAGATAGCCGCCTGGTTGTAG-3’ |
| β-AS-F | 5’-GTTGGTCGTCTCCCACAATCAC-3’ |
| β-AS-R | 5’-CCAGCAGTGACTCCCTAAACCA-3’ |
| β-A28O-F | 5’-CAAGGCATAGGGTTTACAACAATC-3’ |
| β-A28O-R | 5’-CTGGACCTGCACCATTCACAT-3’ |
| UGT1-F | 5’-TAAATCAACCACTATCCCAGCAA-3’ |
| UGT1-R | 5’-CAGTCACTTACTGCCTCGGATACT-3’ |
| UGT2-F | 5’-AGAGCGTGTGGTGTGGGGT-3’ |
| UGT2-R | 5’-CACCGTTCTGAAATCCCTCCTAT-3’ |
| UGT3-F | 5’-CTTATGCCGACTCTCTCGCTTC-3’ |
| UGT3-R | 5’-AGTCCAATGAGGGGAGGGTTTA-3’ |
| UGT4-F | 5’-CCCACAATGAGTCACGAATCC-3’ |
| UGT4-R | 5’-TTCATTTGGGTAATAAGGAAAGTGA-3’ |
| UGT5-F | 5’-CTTCCTGGCTCTATTTCTTCGG-3’ |
| UGT5-R | 5’-TTGGGAGTGTTGGTGAATAAGGA-3’ |
| UGT6-F | 5’-AGTGCCTTGGCTCTGTTCCTTA-3’ |
| UGT6-R | 5’-GATGTTTGGAGGAATGGGGTT-3’ |
| GAPDH-F | 5’-CAGGGAGGCTTTTAGTTCAGGT-3’ |
| GAPDH-R | 5’-ATCACATCTACACCCCTCCAGC-3’ |
