# Supplementary material for: Candidate Genes Involved in the Biosynthesis of Triterpenoid Saponins in Platycodon grandiflorum Identified by Transcriptome Analysis
Source: Front Plant Sci. 2016 May 19;7:673. doi: 10.3389/fpls.2016.00673 (PMC4871891; doi:10.3389/fpls.2016.00673)
Supplement: Supplementary file 4 [file Table_4.DOC]

**Additional file 4. Chemical structures of triterpenoid saponins from the root of *Platycodon grandiflorum***

| Saponins | Aglycone | R_1_ | R_2_ |
| --- | --- | --- | --- |
| Platycodin D | Platycodigenin | Glc- | Api-(1→3)-Xyl-(1→4)-Rha-(1→2)-Ara- |
| Platycodin D_2_ |  | Lam- | Api-(1→3)-Xyl-(1→4)-Rha-(1→2)-Ara- |
| Platycodin D_3_ |  | Gen- | Api-(1→3)-Xyl-(1→4)-Rha-(1→2)-Ara- |
| Deapioplatycodin D |  | Glc- | Xyl-(1→4)-Rha-(1→2)-Ara- |
| Deapioplatycodin D_2_ |  | Lam- | Xyl-(1→4)-Rha-(1→2)-Ara- |
| polygalacin D | Polygalacic acid | Glc- | Api-(1→3)-Xyl-(1→4)-Rha-(1→2)-Ara- |
| platyconic acid A | Platycogenic acid A | Glc- | Api-(1→3)-Xyl-(1→4)-Rha-(1→2)-Ara- |
